# Unreported cases in the 2014-2016 Ebola epidemic: Spatiotemporal variation, and implications for estimating transmission

**DOI:** 10.1371/journal.pntd.0006161

**Published:** 2018-01-22

**Authors:** Benjamin D. Dalziel, Max S. Y. Lau, Amanda Tiffany, Amanda McClelland, Jon Zelner, Jessica R. Bliss, Bryan T. Grenfell

**Affiliations:** 1 Department of Integrative Biology, Oregon State University, Corvallis, Oregon, United States of America; 2 Department of Mathematics, Oregon State University, Corvallis, Oregon, United States of America; 3 Department of Ecology and Evolutionary Biology, Princeton University, Princeton, New Jersey, United States of America; 4 Epidemiology and Population Health, Epicentre, Geneva, Switzerland; 5 Emergency Health, International Federation of Red Cross and Red Crescent Societies, Geneva, Switzerland; 6 Department of Epidemiology and Center for Social Epidemiology and Population Health, University of Michigan, Ann Arbor Michigan, United States of America; 7 The Woodrow Wilson School of Public and International Affairs Princeton University, Princeton, New Jersey, United States of America; Institute for Disease Modeling, UNITED STATES

## Abstract

In the recent 2014–2016 Ebola epidemic in West Africa, non-hospitalized cases were an important component of the chain of transmission. However, non-hospitalized cases are at increased risk of going unreported because of barriers to access to healthcare. Furthermore, underreporting rates may fluctuate over space and time, biasing estimates of disease transmission rates, which are important for understanding spread and planning control measures. We performed a retrospective analysis on community deaths during the recent Ebola epidemic in Sierra Leone to estimate the number of unreported non-hospitalized cases, and to quantify how Ebola reporting rates varied across locations and over time. We then tested if variation in reporting rates affected the estimates of disease transmission rates that were used in surveillance and response. We found significant variation in reporting rates among districts, and district-specific rates of increase in reporting over time. Correcting time series of numbers of cases for variable reporting rates led, in some instances, to different estimates of the time-varying reproduction number of the epidemic, particularly outside the capital. Future analyses that compare Ebola transmission rates over time and across locations may be improved by considering the impacts of differential reporting rates.

## Author summary

Epidemics are defined by a surge of cases of a disease, yet often a significant number of cases in an epidemic are never reported, for example because not all infected individuals have access to medical care. This underreporting can introduce bias into analyses of disease spread, by distorting patterns in where and when the most cases are observed. Conversely, quantifying underreporting can improve epidemic forecasts and containment strategies. In this study, we analyze data from the recent Ebola epidemic in West Africa, including the time, location and Ebola status of 6491 individual community burials, conducted over 25 weeks in four districts in Sierra Leone. We quantify how reporting rates varied over space and time, and show that estimates of transmission rates that are corrected for dynamic underreporting diverge significantly from uncorrected estimates, particularly earlier in the epidemic and outside the capital.

## Introduction

Despite the unprecedented scale of the 2014–2016 Ebola epidemic in West Africa [1], significant uncertainty remains about the precise number of cases involved, and about the spatiotemporal dynamics of transmission [2–8]. The numbers of non-hospitalized, community-based cases over time and among locations, are particularly uncertain. Ebola cases who become sick and die in the community are at increased risk of onward transmission, with caregiving and fluid contact as especially important transmission routes [9–11]. Accurate case counts over space and time, which include non-hospitalized cases, are important for estimating disease transmission rates, and identifying response strategies [6,12–14].

A central problem is that non-hospitalized cases are less likely to be reported, compared to hospitalized cases: the WHO 2014–2016 Ebola case line-list predominantly includes cases who have been hospitalized [15]. As a result, non-hospitalized cases are underrepresented in Ebola surveillance data, and the observed pattern of cases over time reflects the dynamics of hospital bed capacity and access to formal care as well as the underlying trajectory of the epidemic [16]. A community case may be detected outside of clinical care through interaction with other epidemic response measures, for instance by inclusion in contact tracing, or by laboratory testing of a specimen collected before or after death. However, during the recent epidemic these measures were implemented heterogeneously, due to constraints on public health systems in the affected countries.

Estimates of the reporting rate—the proportion of the total number of infected individuals over a specified time period that are reported as cases—range from 0.33 [17] to 0.83 [18], which bound the initial estimate by the United States Centers for Disease Control (CDC) of 0.40 [19]. The differences among these estimates may stem in part from the different analytical methods used, including capture recapture methods [17], inference from viral sequence data [18] and comparison of hospitalized cases with the projections of a compartmental epidemic model [19].

Reporting rates may vary significantly over time and location. For instance, temporal fluctuations in the number of available hospital beds may cause corresponding fluctuations in case ascertainment rates [11]. Increased case ascertainment through increased access to care may itself lower transmission rates, because hospitalized cases are less likely to transmit [10]. The number of non-hospitalized cases, and the proportion of non-hospitalized cases relative to the total case burden, are also important indicators of the impact of interventions [20], as fewer community based cases reflects better access to hospital care, or a downturn in transmission rates, or both. Estimates of reporting rates for non-hospitalized cases may not be a priority at the beginning of an outbreak. During an outbreak, such infrastructure is difficult to establish at an appropriate scale, beyond informal reports, until resources are allocated according to case data from patient hospitalizations. As a result, the non-hospitalized cases that occur in the initial weeks of an outbreak, in a new area, are often not extensively captured. Thus, transmission and reporting may be intertwined, making it difficult to tell whether a change in incidence is driven by contagion or surveillance thereof, although the relative contributions of the two processes can have important implications for public health responses. Estimates of disease transmission dynamics during the 2014–2016 Ebola epidemic drove recommendations for a variety of containment strategies—including contact tracing, quarantine, and safe burial—based on estimates of the reproductive number of the disease in different contexts [21]. However, it is unclear how robust these analyses are to temporal and spatial fluctuations in ascertainment bias in case time series, especially in situations where many cases are unreported.

Here we disentangle reporting and epidemic dynamics for the recent Ebola epidemic in Sierra Leone using individual-level records of burials performed in the Safe and Dignified Burial (SDB) program that was coordinated by the International Federation of Red Cross and Red Crescent Societies (IFRC) in Sierra Leone. Safe and dignified burial was required by law for every community death in Sierra Leone, during the timespan of the data analyzed here (Oct 20, 2014 –March 30, 2015). Safe burials were to be conducted in the same way regardless of the suspected cause of death, including collecting a skin swab sample for Ebola testing [22]. However, in practice safe burial was not conducted for every community death, and not all community deaths were tested for Ebola. Non-hospitalized Ebola deaths that did not interface with the SDB program, or where a swab sample was not tested, represent a primary source of underreporting in the epidemic, as well as a significant potential source of onward transmission.

Below we describe how the prevalence of Ebola in burial swab samples can be used to estimate the total number of non-hospitalized cases, which can in turn be used to estimate location- and time-specific reporting rates. Using these data, we examine how reporting rates varied over time and across districts, and reconstruct the epidemic curves in each district, accounting for unreported cases. Finally, we address the question of how spatiotemporal dynamics in reporting might affect estimates of disease transmission rates. Our results agree with previous studies [17–19] showing that a substantial fraction of Ebola cases in the 2014–2015 epidemic were unreported. In addition, we find substantial systematic variation across geographic regions and over time in the level of underreporting. In some cases, this variation was sufficient to systematically alter estimates of the reproduction number of the epidemic.

## Methods

### Ethics statement

This study relies solely on retrospective analysis of de-identified data, which was collected as part of a humanitarian response and not for research purposes, and is exempt from ethics committee approval.

### Data

#### Number of non-hospitalized deaths and Ebola prevalence in the SDB program

We used IFRC SDB data on the time and location of 6491 individual burials, conducted over 25 weeks, between Oct 17, 2014 and April 3, 2015 in four districts in Sierra Leone: the capital district of Western Area Urban, the adjacent district of Western Area Rural, and the districts of Bo and Bombali. Data for 4020 (61.9%) of the burials included the results of laboratory testing of a skin swab sample for Ebola. In this subset, 386 (9.6%) burials tested positive for Ebola. These data were available for each district and week.

#### Population size and weekly all cause community mortality

Estimates of population size in each district were taken from the 2015 census [23]. Estimates of weekly all-cause community mortality were taken from a household survey conducted in the capital district of Western Area Urban in 2015 [24]. This survey reported raw all-cause mortality of 36 deaths at home over a recall period of 267 days for 898 households, with a mean household size of 6.66 individuals. This yields an all-cause mortality rate of 8.22 deaths at home per thousand population per year. This was converted to expected deaths per week in each district by rescaling the time units, and multiplying by the estimated population size of the district. This approach, designed to work with available recent data, makes the necessary assumption that community mortality rates in each district in the study are similar to that observed in the capital district.

### Analysis

#### Estimating the number of unreported Ebola cases, and reporting rate over space and time

Let *c* represent the number of unreported Ebola cases in a given week and district, and *W* the number of reported cases. *W* was assembled from the weekly situation reports of confirmed and probable cases given by the World Health Organization (WHO) [25]. The weekly, district-specific reporting rate is then

$$\rho =\frac{W}{c+W}$$ (1)

where the denominator represents the total number of cases, both reported and unreported.

We are interested in unreported cases, *c*, but the SDB data consists entirely of deaths, some of which were Ebola positive, and some of which were reported. We first setup a framework for converting from unreported Ebola deaths to unreported Ebola cases, then describe how we use the SDB data to estimate unreported Ebola deaths. Let *d* represent the number of unreported deaths, which is related to the number of unreported cases by *d* = *γc*, where *γ* is the fraction of unreported cases in a week that die in that week (and where the death is also unreported). For the average Ebola fatality in the recent epidemic, death occurred within approximately two days of WHO notification, if the case was reported [26]. Furthermore, *c* must approach *d* as case fatality rate (CFR) increases. We therefore assume that *γ* is equal to the CFR for Ebola. This approach introduces uncertainties into the analysis: for example, a death may be more likely to be reported than a case, and estimates of CFR for Ebola are wide-ranging, and may vary systematically with different contexts [27]. To address these uncertainties, we used a wide range of values for *γ*, specifically the set {0.25, 0.5, 0.9}.

**Fig 1:**
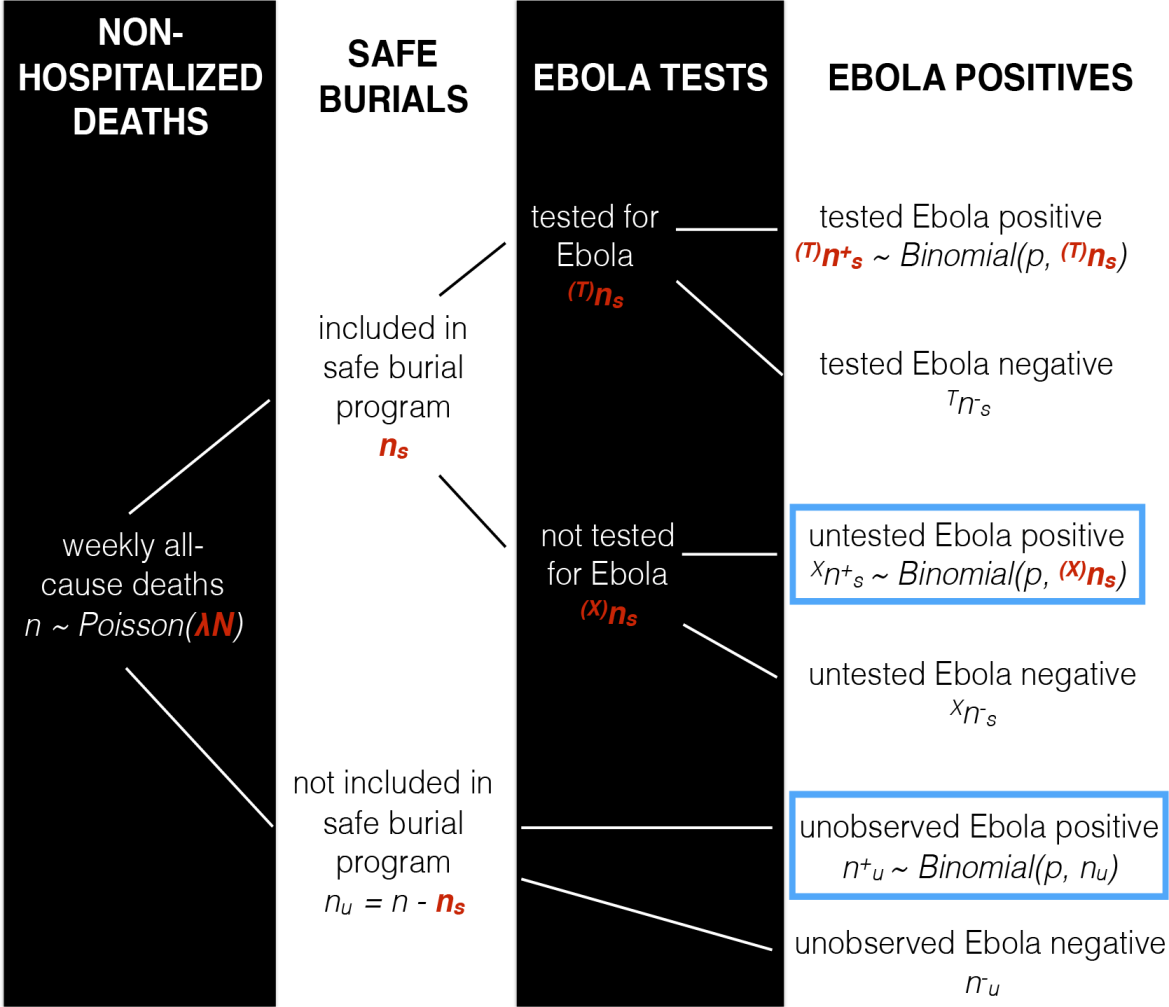
Hierarchical binomial tree for classifying reported and unreported non-hospitalized Ebola deaths. Variables highlighted in red are taken from data. Blue squares indicate the components of the total unreported community Ebola deaths per week.

Now that we have a method of converting from unreported deaths to unreported cases, we continue by estimating unreported deaths from the SDB data. Community deaths overall can be classified using a dichotomous tree, reflecting their interaction with the SDB program and their Ebola status (Fig 1). The structure of the tree makes it possible to estimate the total unreported non-hospitalized Ebola deaths in a district and week, *d*, based upon the corresponding number of Ebola positive and negative burials conducted by the SDB program, combined with estimates of background mortality.

Let *λ* represent the weekly per-capita rate of all-cause community mortality in a district. The corresponding number of deaths in a given week is a stochastic process that can be modeled as a Poisson process

n∼Poisson(λN) (2)

where *N* is the population size of the district, *n* is the number of non-hospitalized deaths in a given week and district, and “*~*” means “distributed as,” indicating the probability distribution for the random variable on the left-hand side of the equation.

In each week and district a number of these deaths *n*_*s*_ ≤ *n* are buried by the SDB program, while the remaining *n*_*u*_ = *n* − *n*_*s*_ are not. Of the burials that are done through the SDB program, a number ^(*T*)^*n*_*s*_ ≤ *n*_*s*_ have a skin swab sample taken for laboratory confirmation of Ebola, while the remaining ^(*X*)^*n*_*s*_ = *n*_*s*_ − ^(*T*)^*n*_*s*_ do not.

The total weekly burden of Ebola-positive non-hospitalized deaths in a district thus has three components: non-hospitalized deaths buried by the SDB program with a positive laboratory result, ${}^{\left(T\right)}n_{s}^{+}$; non-hospitalized deaths buried by the SDB program that were Ebola positive but not tested, ${}^{\left(X\right)}n_{s}^{+}$; and non-hospitalized deaths that were not buried by the SDB program that were nonetheless Ebola positive, $n_{u}^{+}$. The first component of the total burden, ${}^{\left(T\right)}n_{s}^{+}$, is observed and assumed to be reported; the other two components are not observed, and are assumed to comprise the number of unreported Ebola deaths, $d={}^{\left(X\right)}n_{s}^{+}+n_{u}^{+}$. We proceed by using the observed component ${}^{\left(T\right)}n_{s}^{+}$ to estimate Ebola *prevalenc*e in non-hospitalized deaths in a given district and week, then use that prevalence to impute the two unobserved components, thus yielding *d*.

The observed number of Ebola-positive SDB burials with a lab result, ${}^{\left(T\right)}n_{s}^{+}$, can be modeled as

$${}^{\left(T\right)}n_{s}^{+}∼Binomial(p,{}^{\left(T\right)}n_{s})$$ (3)

where *p* is the overall weekly prevalence of Ebola in non-hospitalized deaths in a given district. We model the change in *p* over time in each district by spline interpolation using a single polynomial function

$$p(t)=\overset{k_{max}}{\underset{k=0}{\sum }}a_{k}t^{k}$$ (4)

where *t* is time in weeks and the parameter set *ϕ* = {*a*_*k*_} captures the temporal dynamics of *p and k*_*max*_ = |*ϕ*| − 1 = 3. This represents a ratio of parameter values to time points in the data of 4:25, which reduces the risk of over-fitting the model, as compared to modeling each week independently. Note that by focusing on the weekly *prevalence* of Ebola in non-hospitalized deaths, the method is unbiased with respect to fluctuations in the total number of burials (both Ebola positive and negative, tested and untested) conducted by the SDB program.

Given data on the number of burials with a laboratory test, and number of positives among those burials, the likelihood of a parameter set *ϕ*, is simply the product, over time points, of binomial densities, as per Eq (3), with probability parameter *p* as a function of time given by Eq (4). It is then straightforward to fit *ϕ* to data from each district using a Bayesian approach, via Markov Chain Monte Carlo (MCMC) sampling. In this analysis, we used vague prior distributions, and achieved operational convergence by repeatedly reinitializing the chain, verifying that the posterior distribution of the parameters did not depend on the initial state of the sampler. When estimating the posterior distribution for *ϕ* in each district we discarded the first 10000 steps of the chain as “burn-in.” We then imputed, conditional on *p*, the two unobserved components of total non-hospitalized Ebola mortality: non-hospitalized deaths buried by the SDB program that were Ebola positive but not tested, ${}^{\left(X\right)}n_{s}^{+}$, and non-hospitalized deaths that were not buried by the SDB program that were Ebola positive, $n_{u}^{+}$. This was done by sampling from Binomial distributions with probability parameters given by *p*, and size parameters ^(*X*)^*n*_*s*_ and *n*_*u*_ respectively (see Fig 1). In the results, we report midline estimates for *ρ*—representing the median of the posterior distribution for unreported cases *c* divided by the median CFR *γ* = 0.5—and credible intervals—extending from the first quartile for the posterior distribution of *c* divided by the most conservative CFR *γ* = 0.9, to the third quartile for the posterior distribution of *c*, divided by the least conservative CFR *γ* = 0.25.

Fluctuations in all-cause non-hospitalized mortality rate could influence our estimates of Ebola reporting rates. For instance, if all-cause mortality was higher outside the capital district (where our estimate of all-cause mortality came from) then our approach will overestimate the number of non-hospitalized Ebola deaths that were buried by the SDB program, and, in turn, underestimate reporting rates. However, as described below, the magnitude of geographic and temporal variation in reporting rate the we observed, where in districts outside the capital reporting rate more than doubled over the course of the epidemic, is unlikely to be driven primarily by fluctuations in background mortality, because these would need to be unrealistically large.

#### Estimating the time-varying reproduction number

We estimated the time-varying reproduction number (*R*_*t*_) in each district by applying the method of Cori et al. [28] to time series of the number of new infectious individuals each day, reconstructed from the weekly WHO case data, using a time window of 7 days, and a vague prior for the values of R consisting of a gamma distribution with a mean and standard deviation of 5. To reconstruct the daily infectious time series we used a Monte Carlo scheme, projecting each case in the WHO data backward to its time of first infectiousness by sampling from a gamma distribution for the time from the appearance of symptoms to reporting, parameterized from IFRC line list data from Ebola Treatment Center (ETC) operations (gamma distribution with mean 5.1 days and standard deviation 3.5 days). Note that the results of this operation are independent and identically distributed over cases, so the resulting daily infectiousness time series did not project significant uncertainty forward into the analysis, due to the large number of independent cases over which the reconstruction method was applied. When estimating *R*_*t*_, we used a gamma distribution with mean 15.3 days and standard deviation 9.1 days for the serial interval of Ebola [26].

## Results and discussion

**Fig 2:**
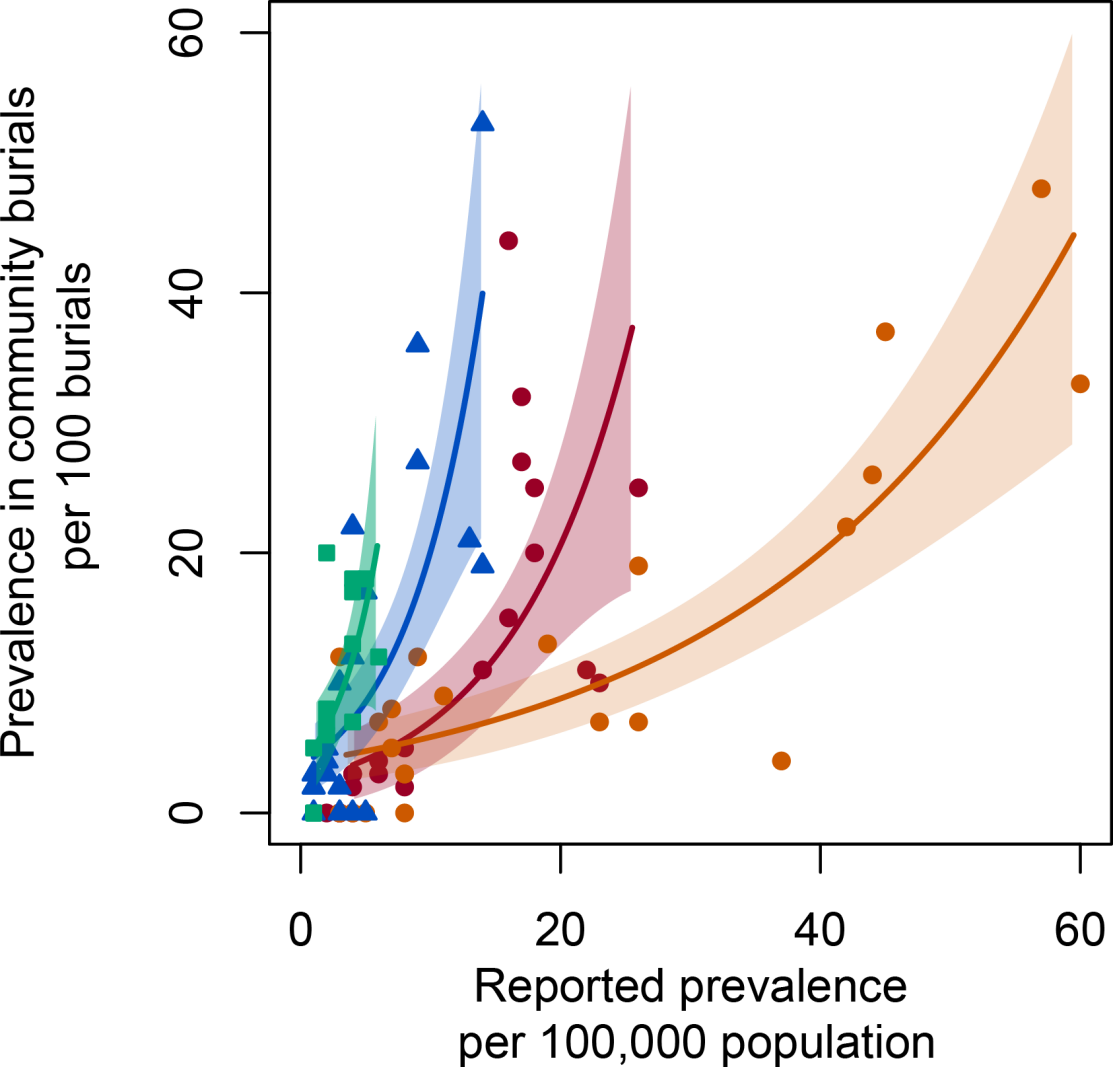
Correlations between reported prevalence in the WHO patient database and reported prevalence in Red Cross burials of non-hospitalized deaths, in four districts of Sierra Leone: Western Area Urban (the capital district; red circles), Western Area Rural (orange circles), Bombali (blue triangles), and Bo (green squares). Polygons enclose +/- 2 standard errors for Poisson regression of non-hospitalized burial prevalence as a function of WHO reported prevalence (confirmed and probable cases) in each district.

**Fig 3:**
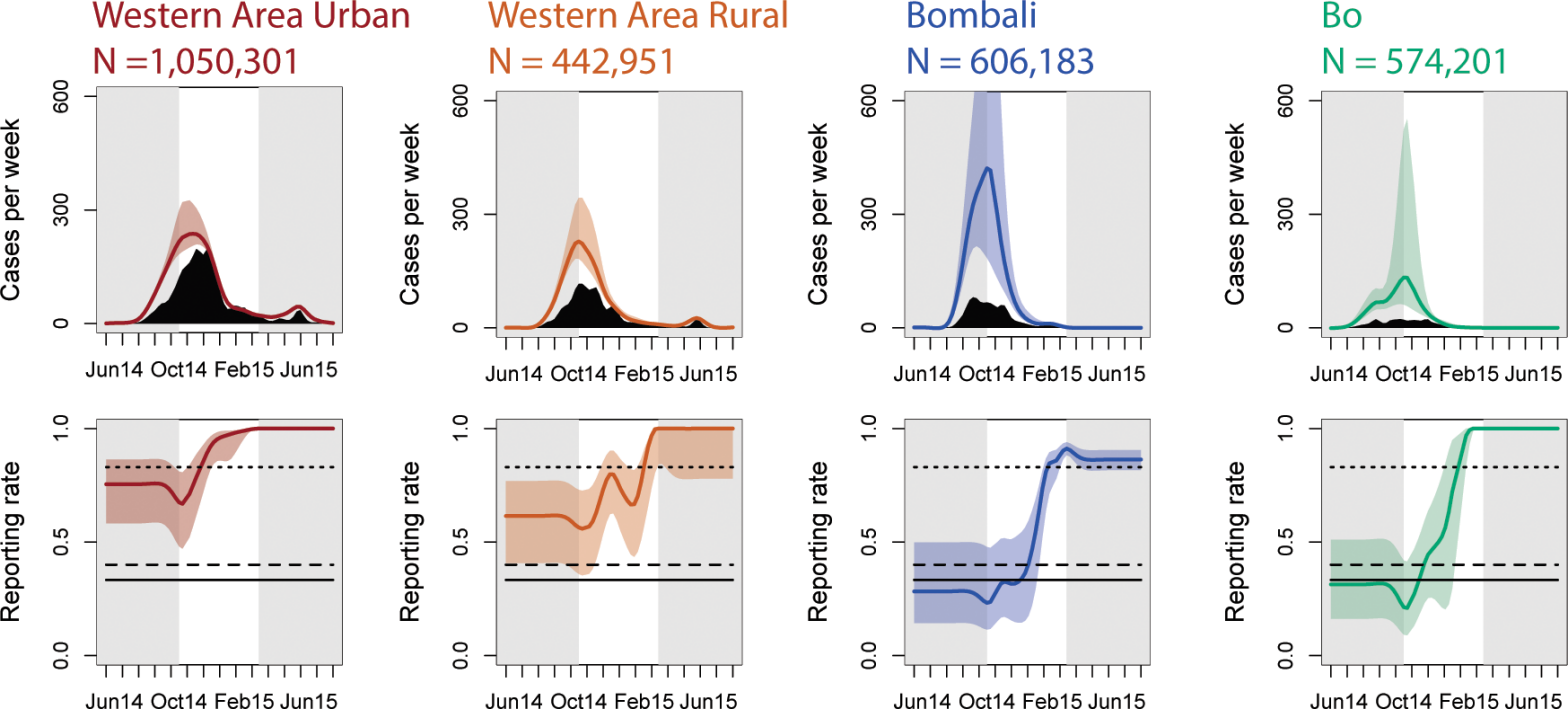
Contrasting estimates of Ebola incidence and reporting rates over space and time. *Upper row*: The heights of the black polygons show weekly numbers of confirmed plus probable cases from the WHO data. Colored polygons enclose credible intervals for total number of cases per week (both reported and unreported). *Lower row*: Estimated reporting rates over time and across districts; polygons enclose the credible intervals. Horizontal lines show static reporting rates estimated by [17] (lower, solid), [19] (middle, dashed) and [18] (top, dotted). The central white region of each plot shows the temporal coverage of the SDB data. To illustrate the temporal coverage of our data relative to the epidemic as a whole, total cases and reporting rates in the grey regions are extrapolated using the mean of the nearest month of burial data. While extrapolated values match published estimates of static reporting rates, these extrapolations are not used in any of the analyses.

Ebola prevalence in the SDB data was strongly correlated with reported prevalence in the WHO situation reports, however, the relationship between burial prevalence and reported prevalence varied across districts (Fig 2, Table 1). In the capital district of Western Area Urban and the adjacent district of Western Area Rural, burial prevalence accrued more slowly under increasing reported prevalence, while in the districts of Bo and Bombali, burial prevalence rose more steeply under increases in reported prevalence. This is consistent with the hypothesis that reported Ebola prevalence outside of the capital was indicative of a proportionally larger number of unreported, non-hospitalized cases (Fig 3, top row).

**Table 1:** Model coefficients and performance for Poisson regression of burial prevalence per 100 burials as a function of reported prevalence (confirmed and probable cases in the WHO data) per 100,000 population.

Coefficient	Estimate	Standard error	p
Reported Prevalence (RP)	0.038495	0.004306	< 10^−6^
Bo (BO)	1.004747	0.148268	< 10^−6^
Bombali (BM)	1.404971	0.119487	< 10^−6^
Western Area Rural (WR)	1.359832	0.125925	< 10^−6^
Western Area Urban (WU)	1.159831	0.148181	< 10^−6^
RP X BM	-0.023126	0.004455	< 10^−6^
RP X WR	-0.034296	0.004316	< 10^−6^
RP X WU	-0.028590	0.004386	< 10^−6^
Null deviance (NUL): 4211.35 Residual deviance (RES): 539.16 R^2^ = 1 –NUL/RES = 0.871974			

Fitting the time-varying hierarchical binomial model to the data quantified variation in reporting rate over time and among districts (Fig 3, bottom row). Midline estimated reporting rate in Western Area Urban on Oct 20, 2014 was 0.68 (credible interval: {0.45, 0.73}). However, estimates for the same date in other districts were significantly lower, at 0.55, 0.27 and 0.33, for Western Area Rural, Bombali, and Bo respectively. Reporting rates increased over the course of the epidemic in all districts in the data, so that by March 30, 2015, reporting rates in all districts were estimated to be at or above Scarpino et al.’s estimate of 0.83 [18]. However, reporting rates outside the capital district stayed lower for longer before beginning to increase. For instance, while the midline estimated reporting rate in Western Area Urban on the week of January 5, 2015 had risen to 0.96 (credible interval: {0.93,0.98}), estimates for the same date in Bombali and Bo remained close to their initial values (see Fig 3, bottom row).

**Fig 4:**
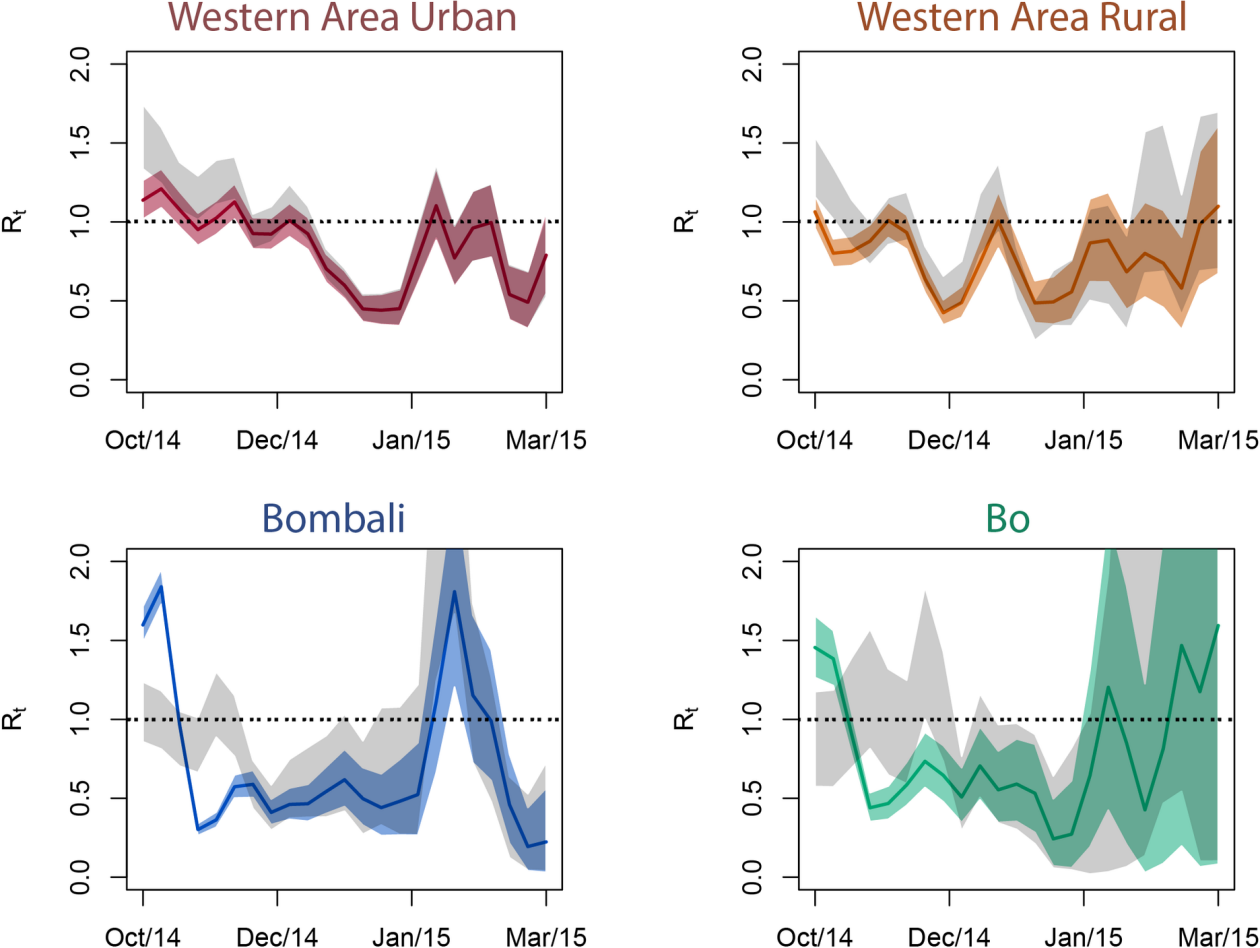
The estimated effective reproduction number of the Ebola epidemic over time in four districts of Sierra Leone, based the WHO data, either corrected for variable underreporting (colors) or uncorrected (grey). Polygons enclose the interquartile range of the credible interval on the estimate over time, encompassing the central 50% of the posterior distribution at each time point[28].

The reproduction number of Ebola estimated from the WHO data varied over time and among districts, as in previous estimates [4]. The transmission rate of Ebola may be particularly variable in a community context, due in part to variation in the relative risk of different transmission routes that may occur in a community setting, particularly care-giving outside of hospital [29]. To quantify the effect of reporting variation on estimates of *R*_*t*_ in Ebola incidence data, we compared results for time series that were corrected for underreporting (using our midline estimates of reporting rate in each district over time) with uncorrected time series (Fig 4). While in the Western Area districts (around the capital of Freetown) correcting for temporally variable underreporting did not significantly change estimates of *R*_*t*_, in Bo and Bombali the corrected estimates diverged significantly from the uncorrected estimates, particularly earlier in the epidemic.

Accounting for variation in reporting can significantly modify our understanding of disease spread and control, but variation in reporting rate over space and time is rarely accounted for during analyses of epidemic dynamics. In particular, most models of Ebola transmission to date have assumed constant reporting rates across space and over time [5,6,12,17–19,21,30,31]. Here we used community-based data on non-hospitalized deaths to infer variation in patterns of reporting, finding significant spatiotemporal variability in case ascertainment. This spatiotemporal variability echoes recently described heterogeneity in transmission patterns in non-hospitalized Ebola data, where the importance of superspreading events was demonstrated [32]. Correcting for these reporting variations improved the accuracy and precision of estimates of transmission patterns.

How do fluctuations in reporting rate influence estimates of the reproductive rate of an epidemic? As the number of cases becomes large, the posterior mean of the estimated reproductive number approaches

$$R_{t}=\frac{\overset{t}{\underset{s=t-\tau +1}{\sum }}\rho_{s}I_{s}}{\overset{t}{\underset{s=t-\tau +1}{\sum }}\overset{t}{\underset{u=1}{\sum }}w_{u}\rho_{t-u}I_{t-u}}$$ (5)

where *τ* is the number of time units over which the reproductive number is assumed constant, *ρ*_*t*_ is the reporting rate at time *t*, *I*_*t*_ is the number infectious at time *t*, and *w*_*t*_ is the generation time distribution of the disease, representing the fraction of secondary cases that originate from the primary case *t* time units after the primary case becomes infectious [28]. If the reporting rate is constant, *ρ*_*t*_ = *ρ*_0_, then reporting does not affect estimates of *R*_*t*_, because the constant *ρ*_0_ cancels out in the numerator and denominator of (5). However, variable reporting rates are confounded with generation time, and shape estimates of R_t_ in the same ways that variation in generation time can [33]. For example, a sharp increase in reporting rate will lead to an overestimate of *R*_*t*_ by inflating the numerator relative to the denominator. In the context of field outbreak response, such an increase in reporting might be caused by increased allocation of resources to contact tracing, or an increase in hospital bed capacity.

Variation in reporting rates can also affect measures of uncertainty in estimates of *R*_*t*_. For large numbers of cases, the coefficient of variation in the estimate is given by

$${CV(R}_{t})=\frac{1}{\overset{t}{\underset{s=t-\tau +1}{\sum }}\rho_{s}I_{s}}$$ (6)

which is equivalent to the standard deviation of the estimated R_t_ divided by its mean, and thus can affect the width of the credible intervals for *R*_*t*_. The denominator of (6) is proportional to the covariance between reporting and incidence over a time interval of length *τ*. Thus, if reporting and incidence covary, the credible interval on estimates of the reproductive number will shrink when corrected for underreporting, all else equal.

Future epidemic models may be improved by incorporating a process-based representation of reporting dynamics. More specifically, future models could treat reporting rate as a state variable, driven by human behaviors associated with both disease spread and public health response, and including inequities in access to medical care. Improving our quantitative understanding of what determines reporting rates could also allow stronger links between field outbreak response teams and modeling teams, which would improve contextualization and understanding of data limitations, with the potential to improve predictive models of epidemics and enhance the design of control measures.

## Supporting information

S1 Checklist STROBE checklist. (PDF)

S1 Fig Estimated total cases versus reported cases per week in each district. The estimated total cases shown is that using the median estimate, with γ = 0.5.
(PDF)

S2 Fig MCMC samples of positive burials over time in each district. Each line represents one of 1000 independent realizations from the posterior distribution of φ.
(TIF)
