# Supplementary figures and images for: Unreported cases in the 2014-2016 Ebola epidemic: Spatiotemporal variation, and implications for estimating transmission
Source: PLoS Negl Trop Dis. 2018 Jan 22;12(1):e0006161. doi: 10.1371/journal.pntd.0006161 (PMC5806896; doi:10.1371/journal.pntd.0006161)

Western Area Urban

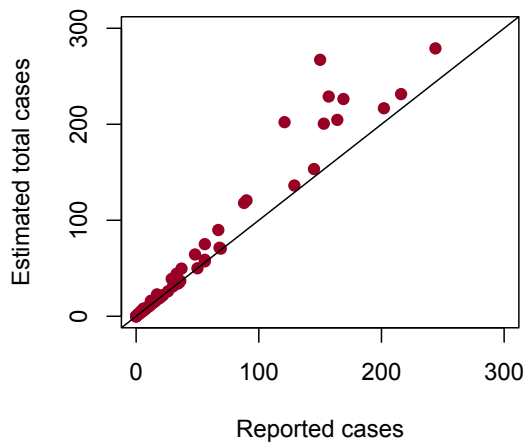

Western Area Rural

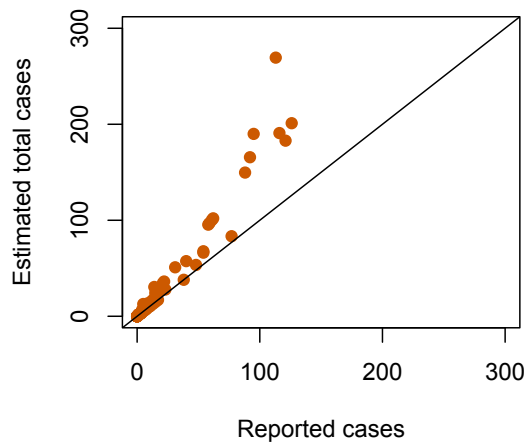

Bombali

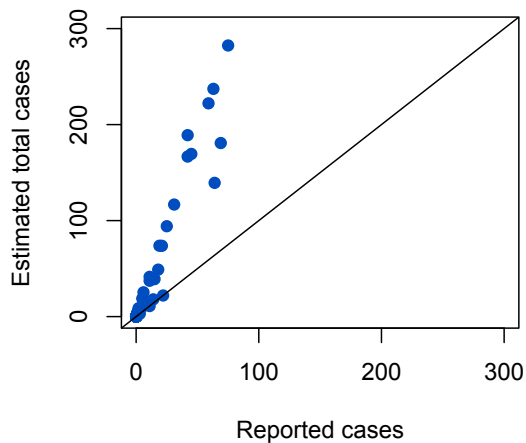

Bo

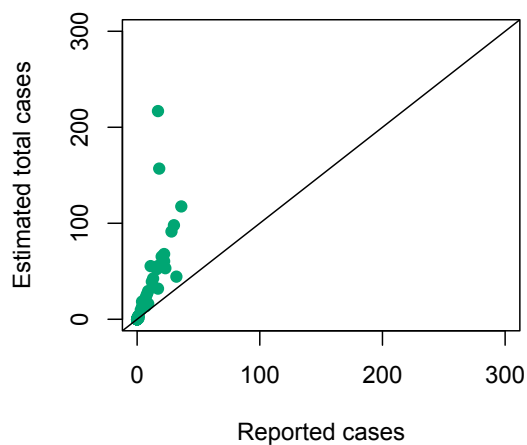

Supplement: S1 Fig — The estimated total cases shown is that using the median estimate, with γ = 0.5. (PDF) [file pntd.0006161.s002.pdf]

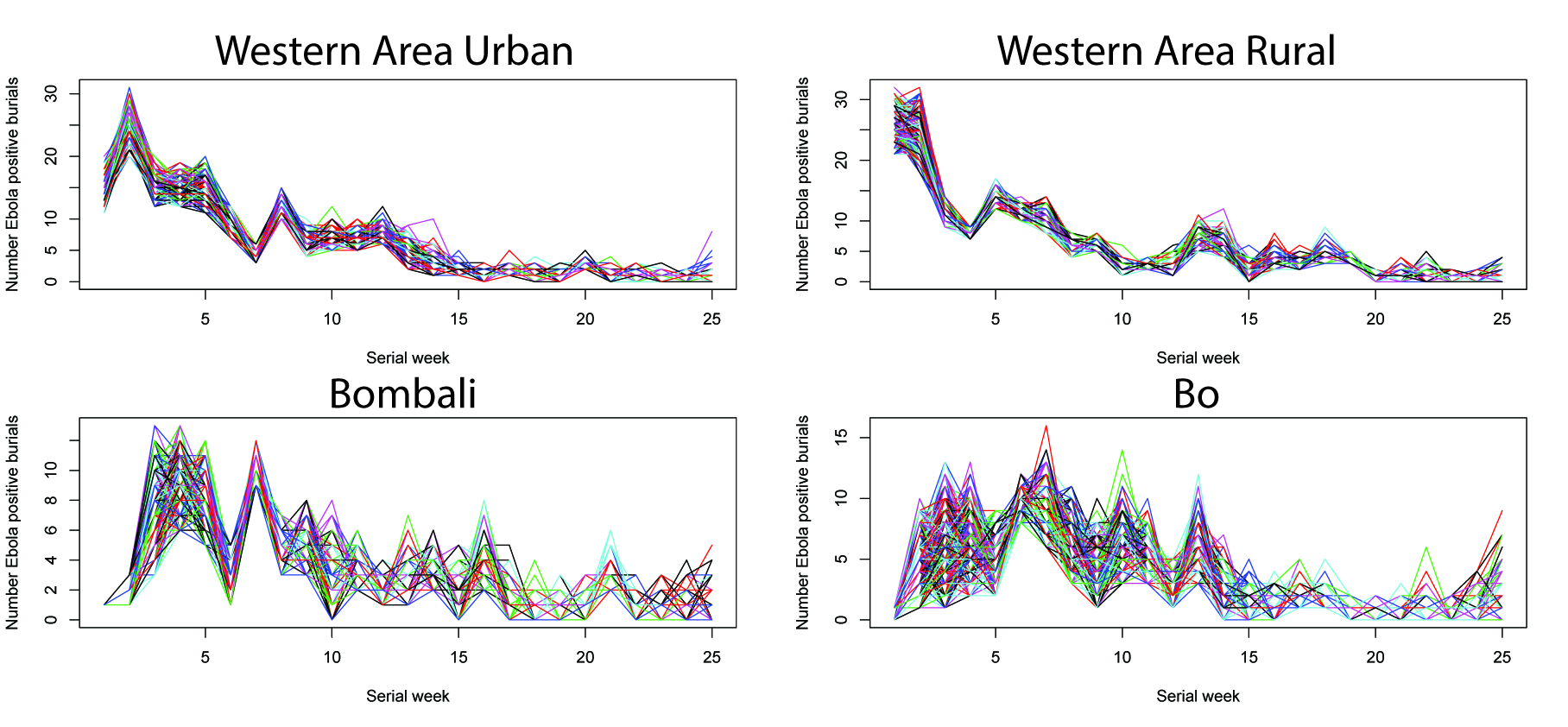

Supplement: S2 Fig — Each line represents one of 1000 independent realizations from the posterior distribution of φ. (TIF) [file pntd.0006161.s003.tif]
